# Understanding Cellular Mechanisms Underlying Airway Epithelial Repair: Selecting the Most Appropriate Animal Models

**DOI:** 10.1100/2012/961684

**Published:** 2012-09-23

**Authors:** B. Yahaya

**Affiliations:** Cluster for Regenerative Medicine, Advanced Medical and Dental Institute (AMDI), Universiti Sains Malaysia, Bandar Putra Bertam, Penang, 13200 Kepala Batas, Malaysia

## Abstract

Understanding the mechanisms underlying the process of regeneration and repair of airway epithelial structures demands close characterization of the associated cellular and molecular events. The choice of an animal model system to study these processes and the role of lung stem cells is debatable since ideally the chosen animal model should offer a valid comparison with the human lung. Species differences may include the complex three-dimensional lung structures, cellular composition of the lung airway as well as transcriptional control of the molecular events in response to airway epithelium regeneration, and repair following injury. In this paper, we discuss issues related to the study of the lung repair and regeneration including the role of putative stem cells in small- and large-animal models. At the end of this paper, the author discuss the potential for using sheep as a model which can help bridge the gap between small-animal model systems and humans.

## 1. Introduction

The lung is a relatively stable organ with low rates of cell turnover, particularly in the airways. With less than 5% of epithelial cells proliferating at any given point in time [1], there appears to be a little need for local self-renewal under normal circumstances. However, following airway epithelial injury, it is clear that the epithelium has great capacity to repair and that the mechanisms involved normally allow for the total restitution of the original complex structure [2, 3]. Understanding the mechanisms underlying the process of regeneration and repair of airway epithelial structures demands close characterization of the associated cellular and molecular events. In the presence of acute epithelial injury, it is recognized that cell proliferation is a feature of repair responses that lead to the resolution of the injury. Evidence suggests that there is rapid repair and resolution following physical injury, at least in small animal models [4–10]. Such models indicate that an initial key event is the propensity of the cells bordering the lesion to dedifferentiate and flatten. These flattened cells then appear to migrate inwards and over the denuded area to restore the barrier function of the epithelium. Proliferation is also a key feature of complex and chronic lung disease. For example, in asthma, airway epithelial cell proliferation varies directly with disease severity [11] whereas the same relationship does not appear to hold for chronic obstructive pulmonary disease (COPD) [12].

In many disease conditions, such as asthma and COPD, where the pathogenesis is believed to involve chronic repetitive airway epithelial injury and inflammation, the damage fails to fully resolve and instead results in tissue remodelling and pathology which may interfere with the function [13]. Key to understanding the factors that divert repair away from resolution and towards remodelling is a close understanding of the dynamic events that operate to resolve epithelial injury.

Whilst virtually all *in vivo* physical injury models of airway epithelial repair are small animal based, it is at least debatable whether these systems offer valid comparisons with similar injury experienced in the human lung. A study of the literature suggests a disparity in the time taken to complete the regeneration of airway epithelium following induced injury. In small animal systems, the majority of the published studies reported that by day seven the epithelium has regained a normal appearance [4–6, 8, 10, 14–17]. In contrast, in the study of Heguy et al., [18] in human subjects, the morphological abnormality of the injured epithelium 7 days after injury was associated with significant changes in gene expression profile—indicating that healing was still underway and normal, in both senses and was achieved by 14 days after injury. This contrast may reflect underlying species differences in the temporal dynamics of the repair process or in the underlying structure and composition of the airway epithelium; however, inherent differences in the extent of injury may also contribute. This is unlikely to be resolved until some of these variables can be aligned between studies and questions probed at a deeper mechanistic level. Preclinical efficacy of stem cell-based therapy still needs a good model that can mimic the human condition. Therefore, a larger animal model, where the cellular composition and lung structure align more closely with the human lung, may prove to be useful in understanding responses to lung injury—both in the context of providing a closer analogue of human airway wall architecture, but also in the context of potentially aligning perturbation and assay techniques. 

## 2. Lung Structure versus Cellular Compositions 

As far as structural considerations are concerned, the human airway epithelium is characterized as pseudostratified columnar and includes ciliated, secretory, and parabasal cells that are linked to a foundation of basal cells that anchors the epithelium to the lamina reticularis [19]. The proximal airways of small mammals, on the other hand, typically are only one or two cells thick and those cells rest on only a very sparse network of basal cells [20, 21]. Notably in the sheep, Mariassy and Plopper [17] quantified eight major cell groups in sheep airway based on their differences in cell morphology, staining properties, and distribution. These cells were mucous cell categories (M1, M2, M3, and M4), ciliated, basal, Clara cells, and serous cells, with the latter being restricted to only the submucosal gland areas [22]. At the level of the most distal airways, Clara and ciliated cells predominated in the epithelial lining, whereas more proximally mucous (M1, M2, M3, and M4) and basal cells shared the epithelial lining [22]. Whilst it is likely that the range of cell types present in the intact epithelium and their properties will have a role in determining the response to injury, there have been limited studies to date that have attempted to accurately define such responses in the context of a large mammalian lung *in vivo* [14, 23, 24]. 

In terms of aligning protocols, since bronchoscopic procedures form the basis of clinical respiratory research, a significant advantage could be gained from animal model systems that can offer the same facility of access, together with the potential of repeated assessment within the same individual [25]. In a study carried out by Evans et al., [21], sheep were used as a large animal model to study the dynamics of airway epithelial repair following physical injury induced by mechanical means. The induction of injury was through equivalent employment of a bronchial brushing technique that was selected on the basis of its widespread use as a routine and safe method for cell sampling in clinical and veterinary clinical respiratory medicine.

## 3. Models of Airway Injury and Repair in the Lung

In the context of characterizing the processes occurring during healing subsequent to injury of the airway epithelium, advantage has been taken of a number of animal model systems which are broadly divided into those systems which rely on physical injury [2, 4–8, 10, 14, 15, 26–28] and those reliant on chemically induced injury—whether administered by inhalation or systemic means [8, 17, 23, 24, 29–34]. The nature and extent of injury vary widely between such model systems.

In this regard, there are four general categories of injury to the airway epithelium. The first is described as being reversible—healing occurs after the irritant is removed and the cells return to normal. The second involves exfoliation of individual cells but leaves the majority of the nonciliated columnar and basal cells intact. The third involves desquamation of the group of cells, but leaves the basal cell layer intact. The fourth category involves desquamation of cells, including the basal cells and after the injury, the repair process involves proliferation and migration of surviving nonciliated cells and differentiation to the normal phenotype, with the degree of proliferation reflecting the amount of injury that occurred. In developing suitable models to study cellular responses involved in airway epithelial regeneration and repair, various methods have been applied which allow targeting the damage to particular regions or different cell types.

## 4. Chemical Induction of Lung Injury

Chemically induced lung injury is well established as a means of achieving airway epithelial perturbation. Such techniques include exposing the lung to high concentrations of oxygen (O_2_) or oxidant gases such as nitrogen dioxide (NO_2_). Cell death from hyperoxic injury may occur through either apoptotic or non-apoptotic pathways, possibly via free oxygen radicals [35]. It is appreciated that the different types of cell present at the air interface in the lung exhibit a range of sensitivity to the effects of chemical exposure. For instance, the alveolar epithelial cell type I (AEC I) is particularly sensitive to bleomycin and the first to be injured in the context of exposure to this chemical, whereas alveolar epithelial cell type II (AEC II) has a more variable sensitivity [36]. Subsets of Clara cells in the conducting airway epithelium are recognized to be differentially sensitive to naphthalene [32, 37]. These susceptibilities should be borne in mind when interpreting the response to injury mediated by these chemicals. 

Whilst the general utility of chemically induced lung injury in small animal models is reflected in their popularity, it is important to reflect upon their comparative relevance. Indeed, naphthalene is a cytochrome P-450-activated Clara cell cytotoxicant that, in mice, causes swelling and vacuolation of Clara cells in terminal bronchioles leading to exfoliation and necrosis of the majority of cells in the airways of the deep lung [38]. Mice appear to be much more susceptible to this toxicant than other species, including nonhuman primates, rats, and hamsters [39, 40] and this fact, coupled with the predominance of this cell type in the proximal conducting airway in mice relative to humans, suggests that the potential exists for mechanisms of repair evoked as a consequence of such injury to vary between species. Indeed, the cellular composition of the tracheal and the tracheobronchial tree of the airway does differ between species—with Clara cells in mice representing 49% of the total epithelial cells in tracheal epithelium, whilst at the same site in monkeys or humans less than 1% of the cells are Clara cells [41]. Further, submucosal glands are important secretory glands that are, present in the major airways and bronchioles of humans, and whilst in mice the structure, cellular composition and density of tracheal submucosal gland (SMG) are similar to those seen in humans, this is in fact the only site where they are seen in this species [42]. Lastly, there are obvious differences between large and small animals in the thickness of the epithelium with the former being characterized by a thicker epithelium. These differences raise the possibility that mechanisms of repair evoked as a consequence of injury may also vary between species [41].

Chemically induced lung injury normally causes relatively mild injury (is this true for injury caused by detergents i.e polidocanol?) involving the shedding of the epithelium whilst leaving an attenuated squamous basal cell layer and underlying tissue architecture [43]. Any remaining or lesion-bordering epithelial cells have the capacity to quickly regenerate a pseudostratified epithelium and both basal and/or nonciliated columnar epithelial cells have the potential to serve as primary progenitor cells in this regard [44, 45]. Although this technique allows for the perturbation of the airway epithelial morphology, the extent and location of the epithelial damage are nonuniform throughout the exposed areas. 

Such injury models coupled with transgenic and cell type-specific knockout strategies in mice have been used to probe the importance of Wnt/beta-catenin signalling pathways in bronchial regeneration and repair in this species [46]. Manipulation of gene-specific cell types also promises the ability to trace the roles of each epithelial cell type (i.e., Clara and alveolar type I and II) in airway epithelial proliferation and differentiation during lung development. This includes studies directed towards determining the role(s) of GATA-6 in regulating bronchioalveolar stem cells (BASCs) during development and airway regeneration [47].

Such chemically induced models of lung injury are not confined to the domain of small animal models. For instance, a model of smoke-induced lung injury in sheep is widely considered to have appropriate comparative relevance to the human [48, 49]. With similarities in terms of the immediate response to direct lung injury, the subsequent inflammatory response syndrome, and similar therapeutic opportunities and application including mechanical ventilation (with associated risk of baro/volutrauma), the model continues to inform. In addition, the use of the goat as a model for smoke-induced lung injury was reported as reliable and of comparative relevance particularly in the context of histopathological changes in the tracheobronchial tree [50]. One such study demonstrated that within 1 day of exposure to toxic smoke, the columnar epithelium sloughed intact from the trachea with a concomitant reduction of nearly 35% in the basal cell population. Complete repair occurred within 18–22 days following injury [23].

It is, however, difficult to align such studies across species wherein the extent of the injury itself, and presumably the evoked mechanisms of repair are a prima facie reflection of fundamental differences in anatomy, dosimetry, and xenobiotic metabolism.

## 5. Physical Methods: Mechanical Trauma

Whilst not immune from the effects of species differences in the anatomy of the tracheobronchial tree, methods that use physical means to perturb the airway epithelium have provided an alternative strategy to study the mechanisms involved in successful repair and regeneration following injury.  Table 1 shows a summary of the mechanical injury techniques used in animal model systems The extent of the injury varied—in some models no epithelial cells remained on the denuded epithelium, whilst in others the basal cell layer was left intact.

Wilhelm [10] was the first to apply a mechanical injury, induced by curettage, to the tracheal airway mucosa in a small-animal (rat) model system. The induced injury was relatively severe—involving the complete removal of the intact mucosa [10]. A subsequent study carried out by Hilding [14] employed the use of cotton swabs to mechanically injure the trachea of calves. In this study, the lesion was produced by passing a weighted cotton swab over the tracheal epithelium and caused only the exfoliation and loss of mature columnar cells, leaving the basal layer intact [14].

A series of studies carried out by Keenan and colleagues [4–6, 15] studied the process of repair and regeneration following mechanical injury applied to the tracheae of hamsters. In these experiments, the ventral surface of the trachea was exposed surgically and incised at the intercartilaginous portion immediately posterior to the larynx. A mechanical injury was induced by a single stroke of a small stainless steel probe along the ventral quadrant of the tracheal mucosa from the carina to the larynx. The basal layer was left intact [4–6]. These studies were similar in principle to a study carried out by Lane and Gordon, in which the dorsal surface of the trachea was lightly stroked by a blunt probe. In this latter study, there was the loss of most of the damaged cells in the area of injury, leaving a single discontinuous layer of basal cells with occasional residual ciliated or goblet cells [27].

In a study of tracheal epithelial regeneration following a mechanical injury in hamsters [28], a severe injury was induced by scraping with a blunt probe. This method lethally damaged all the epithelial cells in focal areas in order to maximize the extent of the regenerative response and to induce the involvement of all cell types during airway epithelial regeneration and repair [28]. Dedifferentiation and migration of the epithelial cells bordering the lesion were clearly described in this study.

Similar processes were studied in rats following a mechanical injury [7]. The epithelium was lethally injured by exposing the ventral surface of the trachea by surgical incision at the intracartilaginous portion below the larynx. A stainless steel probe was inserted through the surgical incision and stroked the ventral surface of the trachea from carina to larynx [7]. No viable cells were present in the area of damage. The transition area between damaged and undamaged epithelium was abrupt with normal and viable epithelial cells seen at the margins of the wounded area. Those cells from the wound margins appeared to flatten and migrate across the wound site to cover the damaged epithelium. Thereafter the migrated cells contributed towards the formation of an initially poorly differentiated multilayered epithelium [7].

In a study carried out by Erjefalt et al., involving *in vivo* restitution of guinea-pig airway epithelium, the epithelium was removed along the dorsal aspect of the trachea by a steel probe. This technique did not cause damage to the underlying, denuded basement membrane. Basal cells remained attached to the basement membrane. It was reported in this study that the epithelial cells at the wound edge flattened and migrated over the denuded basement membrane within 15 minutes. Cells underwent dedifferentiation with ciliated cells losing their cilia and secretory cells displaying an intense granule discharge at the wound edge [2].

**Figure 1:**
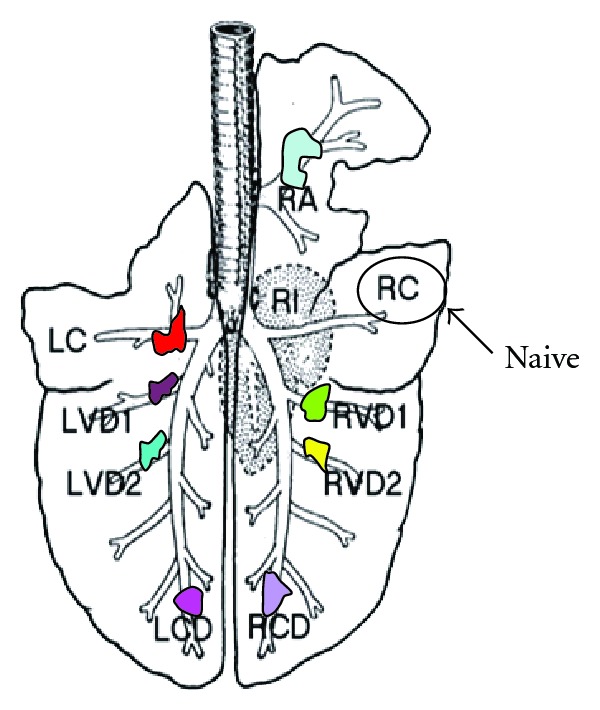
Schematic diagram of the sheep lung with different compartments that can be manipulated to induce injury at different time points. Different colours represent different time point of endobronchial brush injuries. (LC: left cranial; LCD: left cranial diaphragmatic; LVD1: left ventral diaphragmatic lobe 1; LVD2: left ventral diaphragmatic lobe 2; RA: right apical; RC: right cranial; RCD: right cranial diaphragmatic; RVD1: right ventral diaphragmatic lobe 1; RVD2: left ventral diaphragmatic lobe 2).

More recently, our group has established the sheep as a large-animal model to study airway epithelium regeneration and repair following mechanical induced injury [25]. This study utilized an endobronchial brush biopsy to provoke epithelial injury. The endobronchial brush biopsy procedure was applied to multiple different individual bronchial bifurcation sites within the same lung at different time points prior to euthanasia. Those different areas, representing different stages of repair, could then be harvested at necropsy and compared (Figure 1). Preliminary studies indicated that the bronchial brush biopsy procedure resulted in an area of epithelial debridement that effectively repaired over the course of the next 14 days. Repair was characterized by evidence of de-differentiation of goblet and ciliated epithelial cells, flattening and migration of cells to cover the site of injury and proliferation of cells both at the wound margins and in the vicinity of the submucosal glands underneath the damaged epithelium. The number and location of proliferating cells varied according to the time after injury.

The aforementioned preclinical studies [18, 51] in particular emphasize the value of being able to accord gene expression profiles with some experimental state—whether by virtue of exposure to cigarette smoke or bronchial brushing injury—and equally suggest the value of being able to measure and adjust for the influence of between- and within-subject variability in gene expression in analyzing experimental effects. Naturally there are limits to the nature and extent of intervention possible in a clinical research setting and to yield information pertinent to more severe pathology, it is necessary to either collect data from relatively poorly controlled clinical case material and/or from relevant animal model systems. In the latter regard, it is important that the system selected is indeed of comparative relevance, offers the same facility to study expression profiles in selected cells over time in the same subject, and is backed up by the necessary reagents and tools to allow a complete characterization of the process in question.

The issue of comparative relevance is hotly debated by every proponent of every animal model system [52–57] and suffice is to say that no particular system is ideal for all situations and that each system generally has meritable comparative aspects, whether in the context of normal lung structure or function, or in relation to response to injury. In relation to the requirement of following expression profiles in selected cell populations in the same individual animals over time, it is clear that such facility is only really provided by larger animals where routine bronchoscopic procedures can be applied. Whilst it is the availability of reagents and tools that has largely fuelled the popularity of the mouse as a model system for investigating the pathogenesis of airway disease—it is inevitable that, with the inexorable progress underway in relation to genome characterization across species, the nature and extent of tools and technologies available for larger animals will similarly increase.

Whilst some large animals have been employed to study the dynamics of repair in the tracheal epithelium, notably in the context of either very mild trauma (in calves [39, 40]) or induced full thickness mucosal defects (in dogs [58] and rabbits [59]), our study defines the patterns of repair that follow injury to the bronchial epithelium in large animals. Previous studies within our group have indicated the utility and comparative relevance of the sheep as an appropriate large-animal model system to study lung disease pathogenesis and follow the response to lung-directed gene delivery [60].

## 6. The Case for the Sheep as a Model of Comparative Relevance

Sheep are docile, easily handled animals that are readily purchased from commercial stock markets. Sheep tolerate routine procedures such as blood sampling very well and do not constitute a particular anaesthetic risk. For these and other reasons, sheep is favored as an experimental animal in surgical science [61]. With specific respect to the respiratory system, sheep lungs are anatomically closer to those of man than are dog, cat, or monkey lungs [62] and when compared to dogs, sheep appear to be better suited as models for human gas exchange [63]. The size of sheep lungs facilitates the application of various techniques available for the analysis of human cardiorespiratory function. Additionally, in sheep, time-sequential analysis of the respiratory tract, including physiological, biopsy, and lavage procedures, is well tolerated and can be repeated at frequent intervals with no procedure-related effects [64]. Thus, dynamic events following natural or experimental respiratory intervention can be followed in this species.

Prior to the study of Halmagyi and Colebatch, negligible data existed in the literature regarding cardiorespiratory function in sheep. The data reported by these authors included tidal volume, gas exchange, pulmonary mechanics, and blood gas and acid-base values for normal anaesthetized sheep [65]. These authors subsequently went on to utilize their experience of lung function assessment in sheep to examine cardiopulmonary dysfunction in sheep in response to various interventions of both physiological and applied interests [66–68]. This approach, whereby measurement protocols and techniques for lung function analysis are developed and refined prior to their use in the study of respiratory disease, has also been followed by Begin and colleagues in an evaluation of asbestosis in sheep [69, 70]. Other groups have developed and used lung function analysis techniques in sheep to examine the airway [71–78], lung inflammatory responses [79–81], lung function during anaesthesia [82, 83], gas exchange of the lung [63, 84], and physiological responses to exercise [85]. Further, and beyond the experience of our own group, sheep is favoured as a comparative model in respiratory research with examples of applications ranging from smoke inhalation injury [23, 24, 29, 30] to the adult respiratory distress syndrome [86], asthma [87], and emphysema [31]. Sheep and hamster have a large number of airway epithelial cell types which secrete acid glycoprotein in the proximal airways and sheep has glands in abundance [22]. With more basal and ciliated cells than small animals, the respiratory epithelium of the sheep is 4-5 times the thickness of that reported for hamsters and rats (8.6 microns in the hamster to 56.8 microns in the sheep) [21] and relatively similar to the height of the epithelium reported in human (40–50 microns) [41].

## 7. Dedifferentiation, Migration, Proliferation, and Redifferentiation: Integral Steps towards Airway Repair 

The process of airway epithelial repair subsequent to physical injury appears to follow well-defined stages. At an early juncture, unaffected epithelial cells bordering the lesion migrate to cover the damaged area. These migrated cells appear to form a multilayered epithelium, a hyperplastic epidermoid metaplasia. Lastly, these cells redifferentiate to reestablish a normal pseudostratified epithelium [28]. Subsequent to mild injury, in which viable basal cells are left on the surface of the damaged area, regeneration is completed by epithelial hyperplasia and epidermoid metaplasia, followed by differentiation [14, 27]. In this latter instance, basal cells were reported to be the primary progenitor cells contributing to the cellular migration and proliferation in response to injury, whereas in the case of more severe injury (with no basal cells left intact), the secretory and nonciliated cells (goblet cell in particular) were thought to be the primary progenitor cells involved in the cellular de-differentiation, migration and redifferentiation.

The common features that seem to characterize airway epithelial repair following physical injury involve the dedifferentiation of cells bordering the lesion, migration of the flattened cells over the wound area, proliferation, and re-differentiation. In particular, various cell types, including basal, ciliated, and secretory cells, appear to possess the capacity to dedifferentiate, to migrate, and to even re-differentiate to give rise to other cell types. Indeed secretory cells have been shown to dedifferentiate and become flattened epithelial cells when seeded in normal tracheal epithelial cell culture and thereafter re-differentiate to normal morphology whilst basal cells were less frequently observed in a similar process [9]. These observations further suggest that potential may exist to manipulate conditions following injury or even in the context of airway disease such that a fully functional airway epithelium can be completely restored. 

**Figure 2:**
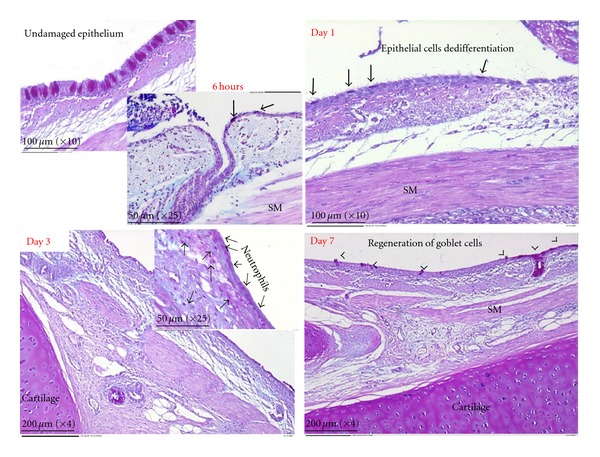
In the normal airways (undamaged), goblet cells were clearly stained with AB-PAS staining (top left panel). At six to twenty-four hours after-injury, PAS-positive cell is no longer retained the characteristic morphology of goblet cells but rather were attenuated in appearance (top right panel; thick arrows) indicating potential epithelial dedifferentiation. By day 3 after-injury, a layer of positive AB-PAS staining was observed overlying the repairing epithelial surface. Closer examination determined that the layer was comprised of a dense and compact accumulation of neutrophils held within and below the mucin layer (bottom left panel; thin arrows). By day 7 after-injury regenerating goblet cells could be seen incorporated in the epithelium overlying the brushed site (bottom right panel; arrows head) an observation taken to imply epithelial redifferentiation was underway. (SM = smooth) [88].

In our sheep bronchial damage model, the time-dependent changes in airway wall morphology were followed by evidence of dedifferentiation in the intact epithelium at the wound margins involving goblet and ciliated cells (Figure 2), followed by proliferation of cells both within the epithelium and in association with the deeper wall structures such as the submucosal glands and smooth muscle. At later time points, there was evidence of redifferentiation in the airway epithelium. These studies demonstrate broad agreement with previous studies in small animals and indicate the general utility of this model as a comparative reference. As such it should provide a solid basis upon which to further characterize the critical cellular and molecular interactions that underlie both effective restitution and pathological repair.

### 7.1. The Role of Stem/Progenitor Cells

Many of the mechanisms that normally operate to engineer tissue repair and which are hypothesised to be overwhelmed in the prelude to lung pathology are believed to have their foundation in lung development. A further aspect of this corollary is that during development, self-renewing tissues are imbued with resident, tissue-specific stem cells, so-called adult somatic stem cells [89]. These stem cells are a subset of relatively undifferentiated cells with the capacity to maintain multipotency in the context of the physiologic domain in which they reside. In this context, adult lung stem cells are capable of abundant self-renewal and regeneration, and should act as stem/progenitor cells in response to injury and effect local repair [35, 90–93]. As such, these cells may serve as a viable target for manipulation in the context of the abnormal repair patterns that threaten normal lung physiology. Several cell types of the lung capable of functioning as stem/progenitor cells in response to injury have been identified; these cells are thought to localize to proximal airway submucosal gland ducts, intercartilaginous ring regions, neuroepithelial bodies, and terminal bronchioles/bronchoalveolar duct junctions [32, 94–96]. The cells identified as progenitor or stem cells in the lung appear to vary according to the lung compartment [89, 91, 92]. Equally, these observations potentially imply that the specific repair mechanisms evoked in response to lung injury will draw upon several sources of stem/progenitor cells according to the nature and extent of the damage. In this regard, it has been suggested that slight or moderate injury will result in resident progenitor cell activation to restore tissue homeostasis, whereas a severe injury and extensive epithelial cell loss will promote stem-mediated repair [97].

Proliferative potential is often taken as a defining characteristic of stem cells [98]. The normally slow rate of lung epithelial turnover is easily accommodated by division, migration, and differentiation of resident and/or mobile stem/progenitor cells [99]. Stripp and Reynolds have described the terminology for the progenitor cells as any cell that has the capacity to proliferate and this terminology is commonly used to indicate a cell that is in the process of cell division or has the potential to enter the cell cycle [100]. In contrast to other tissues such as the gut and skin where the epithelium renews on a frequent basis, the murine lung epithelium has little or no turnover in the absence of exogenous stimuli [99]. Therefore, under such conditions, it is difficult to capture the nature of the specific events involved in renewal, and it is only when normal tissue homeostasis are stressed by the need to engineer widespread tissue repair that the relative importance of different cell types within the tissue can be ascertained. It is possible that lung stem cell populations may be a subset of the same cells that function daily as contributors to the gas-exchange machinery, and their stem cell characteristics may only be realized in times when injury of more specialized, or more differentiated, cells occurs [99]. For this reason, several lung injury models have been developed to probe the mechanisms involved in tissue inflammation and repair, and will likely continue as the most useful approaches to identifying stem cell populations and understanding the regulation of stem cell fates in the lung [35]. These include radiation [101], sulphur dioxide (SO_2_), polidocanol [96], bleomycin [36], and naphthalene [95] as well as the previously discussed mechanically induced lung injury [4–7, 10, 15, 16, 27, 28, 102].

The maintenance and fate of the stem cell populations are regulated by their local anatomical and chemical microenvironment or niche. [35, 103]. Because stem cells divide infrequently under normal conditions, DNA-labelling techniques with detectable nucleotide analogues have been employed to track putative stem cells *in situ*. BrdU (5-bromo-2-deoxyuridine) is one such common nucleotide analogue that is classically used to track label-retaining cells (LRCs) after a prolonged “washout” period that dilutes the label within the more rapidly cycling transient-amplifying (TA) cells—committed progenitors derived from stem cells whose propensity for rapid cycling has a defined capacity [104].

### 7.2. Tools and Resources to Study Airway Epithelial Repair

The complexity of the airway wall tissue architecture, the multiple cell lineages involved, and their physical, autocrine, and paracrine interactions have contributed to difficulties and delay in understanding the mechanisms involved during airway regeneration and repair. Accepting the likelihood that cells, genes, and regulatory pathways involved during lung morphogenesis and development may also play a crucial role during lung injury and repair; it is conceivable that key default pathways do indeed exist and that these pathways are evoked in response to acute injury. Further, it is only when such pathways are perturbed or overwhelmed that the sequences of events result in pathology. To get a wider perspective of the genes involved during airway repair, global gene expression profiling has emerged as the ultimate investigators' tool in that it has the capability to screen the expression level of several thousands of genes in each individual sample. Such screening raises the potential that individual genes or gene regulatory pathways with key roles in the repair process can be identified and, in future, that such identification will lead to more informed therapeutic targeting in the context of complex chronic lung diseases. Whilst the past decade has seen the development of such high throughput methods to collect proteomic, genomic, and transcriptomic data, and the increasing application of systems biology approaches to understand biological processes, there has, to our knowledge, been limited attempts to marry these resources to the well-characterized process of airway epithelial repair following physical injury.

The use of this high-throughput technology has been fundamental to identifying genes with key roles in the progression of diseases such as asthma [39, 40], lung cancer [105], and COPD [106] and the promise remains that new biomarkers will be detected and diseases better understood through subclassification according to their underlying mechanisms [106]—in turn adding leverage to genomewide disease association studies. 

Gene expression profiling continues to play a vital role in the context of understanding the response of animal model systems to experimental manipulation. Such studies have encompassed the response of the lung to injury. As a recent example, in a study of gene expression profiling in the lungs of wild-type mice exposed to naphthalene at various time points, 3,552 genes were significantly upregulated and 1,199 genes significantly downregulated following injury [107]. Analysis of gene expression in the repairing lung revealed prominent clusters of upregulated genes with putative roles in regulation of the extracellular matrix and cellular proliferation [107]. It was also suggested that the extracellular matrix (ECM) is dynamically remodelled in response to selective epithelial cell injury and that this process is activated without resolution in the setting of defective airway epithelial repair [107]. Other examples of such expression profiling in the context of airway epithelial injury in small animals relate to assessment of the alterations in the transcriptional program of the lung in a mouse model of acute lung injury (ALI) induced by *E. coli* lipopolysaccharide (LPS) [108], acute respiratory allergen exposure of transgenic BALB/c mice by a single intratracheal aspiration exposure to *Metarhizium anisopliae* crude antigen (*MA*CA) [109], the gene expression profile of mice exposed to cigarette smoke [110], and co-exposure of mice to cigarette smoke, lipopolysaccharide, or smoke plus lipopolysaccharide by inhalation [111].

In a clinical study, using fiber optic bronchoscopy and brushing to sample the human bronchial epithelium, Heguy et al., [18] investigated the modification of gene expression in response to cigarette smoking. Such transcriptomic screening revealed that in comparing between phenotypically normal smokers (no symptoms, normal lung function, and normal imaging) and nonsmokers, microarray analysis of gene expression of the small airway epithelium demonstrated up- and downregulation of genes in multiple categories relevant to the pathogenesis of COPD, including genes coding for cytokines/innate immunity, apoptosis, mucin, response to oxidants and xenobiotics, and general cellular processes [51]. Such studies highlight the depth of information that can exist behind the screen of apparently similar phenotypes and augers well for the future application of such technology in predicting disease onset and hence directing appropriate prophylactic and/or therapeutic strategies.

Another clinical study that relates to our interest in understanding the molecular regulation of gene expression during airway regeneration and repair is a study carried out by Heguy et al., [18] to identify genes participating in the airway epithelial repair of healthy volunteers after exposure to mechanical injury induced by bronchial brushing via fiber optic bronchoscopy. This study revealed that 1,196 genes were significantly differentially expressed at day 7 after injury when compared with day 0 (uninjured) [18]. Among these genes, 60% were associated with the G_2_ and M phases of the cell cycle, and it was suggested that this reflected the fact that most of the cells at this stage of repair had entered the late stage of cell division.

In conclusion, the study conducted by Yahaya et al., [25] elected to employ the clinical technique of bronchial brushing to selectively damage small areas of epithelium and thereby stimulate repair processes in those areas. This technique demonstrates the utility of a novel model of airway epithelial injury in a large-animal model system that can be used to follow the repair process. The model demonstrated changes in airway architecture and histopathology largely consistent with previous studies in small-animal systems and with similar studies conducted in man. The processes of dedifferentiation, migration, proliferation, and redifferentiation were easily characterized and assessed by selectively injuring areas of the airway epithelium. The model further substantiates the notion that the airway epithelium demonstrates a remarkable propensity to repair itself in the event of acute injury. Indeed, although the brushing technique used in that study destroyed most of the morphology of the mucosal region, the ability of the airway to regenerate a new partly differentiated epithelium and other airway components such as smooth muscle and glands as early as 3 to 7 days after injury were remarkable. The study conducted by Yahaya et al., [25] also suggested the potential of experimental protocols involving segmental challenges to maximize data yield whilst minimizing the ethical costs associated with particular questions relating to airway epithelial repair. Therefore, with all issues discussed here, it is believed that the sheep could be a potential stepping stone to fill in the gap between stem cell fantasy and its reality before it can be translated into the human clinical application.

Certain limitations exist that confound the value of such large-animal systems in agricultural species. Notably very few antibodies are available to label cells of interest in order to trace their function and roles during the repair process. As a consequence, our study sought to define the molecular characteristics of the repair process. Towards this end semi-quantitative analysis of reverse transcriptase-polymerase chain reaction (RT-PCR) assays were developed to probe expression profiles of genes that are believed to associate with specific cell types such as basal and ciliated cells (unpublished data). The genes that were considered in this way were beta-catenin, beta-tubulin, keratins 5 and 14, and FoxJ1–cell-specific genes that have been widely reported to involve in airway epithelial regeneration and repair. Our preliminary analysis revealed that whilst the expression profiles of the studied genes did indeed vary over the course of the repair process the extent of within- and between-animal variability in this regard would likely erode the potential ability of this approach to glean clear and definitive statements regarding the likely pathways and mechanisms underlying the different stages of repair (unpublished data). Such ability would of course further be influenced by the nature and extent of gene selection hinged as it was and would be, on the available literature, but as a consequence biased and less likely to pick up species-specific differences. Thoughts have now turned to the potential utility of microarray expression profiling using sheep microarrays made recently commercially available as a means of characterizing the mechanisms underlying the repair process.

The wider relevance of using sheep as a model to study abnormal airway epithelial repair in the context of airway pathology is observed in asthma, COPD, and cystic fibrosis. The nature of the pathogenesis associated with these diseases which share a common theme in that repetitive insult is believed to occur. How such repetitive insult impacts on the normal repair process is currently unknown but is a question that would lend itself well to the model system developed [25]. In particular, it would be possible to re-brush epithelium at different phases of the repair process to examine the impact of such an approach on the underlying gene expression and evoked pathways. Perturbing the normal process of repair and regeneration by repeating (perhaps on several occasions) the brushing process on the repairing epithelium would facilitate the study of the way in which such repeated insult influences the normal transcriptional pattern of repair. Indeed it may be possible to identify the pathways that continue to operate (in time and extent) on the basis of the original injury, those that emerge as unique to the chronic injury and those that deviate from their anticipated pattern or time course of regulation initiated on the basis of the original injury. Such a model has not hitherto been characterized at the molecular level—although the idea of repeated injury has, as previously discussed [112], been followed through to investigate cell cycle in small-animal models. Such studies would have real relevance to understanding fundamental processes in complex diseases and potentially provide therapeutic directions that could be exploited in the future.

## Figures and Tables

**Table 1:** Animal models of mechanical injury. Variation in the severity of injury can be expected to influence the timing of the repair process.

Models	Damage site	Tools	Effect on epithelium	Severity	References
Rat	Tracheae	Curettage	Complete removal of intact mucosa	Severe	[10]
Calf	Tracheae	Cotton swabs	Basal layer retained	Mild	[14]
Hamster	Tracheae	Blunt probe	Basal layer retained	Mild	[4–6, 15]
Rat	Tracheae	Blunt probe	Basal layer retained	Mild	[27]
Hamster	Tracheae	Blunt probe	Complete removal of intact mucosa	Severe	[28]
Rat	Tracheae	Blunt probe	Complete removal of intact mucosa	Severe	[7]
Guinea pig	Tracheae	Steel probe	Scattered basal cells remained attached to the BM	Mild	[2]
Guinea pig	Tracheae	Flattened scrapper	Complete removal of intact mucosa	Severe	[8]
Sheep	Bronchi	Endobronchial brushing	Complete removal of intact mucosa and submucosal regions	Severe	[25]
